# Nutrient and Food Group Intakes among U.S. Children (2–5 Years) Differ by Family Income to Poverty Ratio, NHANES 2011–2018

**DOI:** 10.3390/ijerph182211938

**Published:** 2021-11-13

**Authors:** Katia Fadeyev, Sayaka Nagao-Sato, Marla Reicks

**Affiliations:** Department of Food Science and Nutrition, University of Minnesota, St. Paul, MN 55304, USA; fadey004@umn.edu (K.F.); ssato@umn.edu (S.N.-S.)

**Keywords:** family income, preschool children, nutrient and food group intake, diet quality

## Abstract

Family income may influence nutrient and food group intakes among preschool children, thus increasing risk of nutrient deficiencies. This study compared nutrient and food group intakes and diet quality among a U.S. sample of children 2–5 years of age by family income to poverty ratio (PIR) based on National Health and Nutrition Examination Survey (2011–2018) data (*n* = 2249). Regression analyses were used to compare dietary intakes based on proxy-reported 24-h dietary recalls and Healthy Eating Index-2015 total scores by low PIR versus mid-high PIR levels adjusted for covariates. PIR levels varied by child race/ethnicity and household reference person’s sex, marital status, and education. More than half of the children in the low PIR group (56%) were reported to have received WIC benefits in the past year. Marginally lower calcium (*p* = 0.008) and lower fiber intakes, lower total HEI-2015 scores, and lower intakes of fruits and whole grain foods (all *p* < 0.007) were observed among children in low PIR households compared to mid-high PIR households. However, iron intakes were higher (*p* = 0.0003) among children in low PIR households compared to mid-high PIR households. Lack of other differences in nutrient and food group intakes may be attributable to WIC benefits.

## 1. Introduction

The preschool years are a vital stage of continued cognitive and physical development after a rapid growth spurt in infancy. Because the preschool years are a period of establishing habits, the promotion of healthy eating behaviors is crucial to ensure appropriate maturation and to create a foundation for healthful dietary patterns in later life. Because the current food environment is changing to include more convenience foods, food groups such as vegetables, fruits, and dairy are becoming displaced by empty-calorie alternatives [1,2].

Previous studies have found that on average, the diet of young children (2–5 years) in the U.S. falls short of dietary recommendations for these food groups and the related nutrients, such as calcium, vitamin D, potassium, and fiber [3,4,5,6,7]. Dairy foods, particularly milk, are the top contributors to calcium and vitamin D intake among preschoolers [8]. Inadequate intakes of both nutrients can lead to poor bone mineralization, causing weak bones and increasing the risk of skeletal deformities. Additionally, the calcium retained during early childhood affects peak bone mass in early adulthood; insufficient intakes could lead to an increased risk of metabolic bone disease and fractures [9,10]. With the introduction of processed foods, a greater concern also exists for inadequate intake of potassium and excessive intake of sodium within the preschool population. Consistent low intakes of potassium and increased sodium consumption lead to an increased risk for the prevalence of high blood pressure. In 2017, the Centers for Disease Control and Prevention (CDC) found 1 in 10 U.S. children, ages 12–19, had elevated blood pressure [11]. Limited data are available regarding the benefits of dietary fiber in early childhood. However, adequate intake of fiber is known to support digestive health and may be protective against metabolic disorders, such as type 2 diabetes [12]. Establishing habits of sufficient intake during childhood may promote the continuation of such behaviors in later life.

Dietary intakes of young children are influenced by multiple factors, including family income. Previous studies have found that low-income preschoolers were more likely to be overweight, iron-deficient, and have lower levels of vitamins A and C, and folate compared to middle/high-income children [13,14]. Several studies have also found that low-income households purchased less fruit, vegetables, dairy, and other nutritious products and more sugar-sweetened beverages than households with higher incomes [15,16,17,18,19]. Such results suggest preschoolers living in a low socioeconomic household may be at a higher risk of nutritional deficiencies and poor health due to food insufficiency. However, a recent analysis of 2013–2016 National Health and Nutrition Examination Survey (NHANES) data showed no differences in dietary intake by three income levels for children ages 2–8 years old [7]. The lack of difference may be the result of WIC participation among lower-income children. Jun et al. [20] found no differences in calcium, iron, zinc, potassium, or fiber intake between participants and non-participants in the Special Supplemental Nutrition Program for Women, Infants and Children (WIC), including those of higher income. Therefore, the impact of family income on the dietary intake of preschool children is not clear. Limited studies have examined relationships between dietary intake of preschool children and family income that supports the capacity to meet dietary recommendations based on recent, nationally representative data. The purpose of this study was to determine potential differences in dietary intake among preschool children (ages 2–5) by two income levels (low and mid-high income status according to the family income to poverty ratio) based on 2011–2018 NHANES data.

## 2. Materials and Methods

### 2.1. Overview

This study was a secondary analysis of existing data from the 2011–2018 NHANES. NHANES is a cross-sectional study that utilizes a sampling method with a multifaceted probability design to produce a nationally representative sample of the U.S. population [21]. The study sample included participants aged 2–5 years (*n* = 3610 with 2 days of 24-h recall interview data and *n* = 2249 with reliable recall data for those 2 days). All participant information was gathered from a proxy. The National Center for Health Statistics (NCHS) Institutional Review Board for the Ethics Review Board approved NHANES data collection [22] with written informed consent from participants before data collection. The University of Minnesota Institutional Review Board determined that analysis of de-identified, publicly available NHANES data was not research involving human subjects as defined by the U.S. Department of Health and Human Services and Food and Drug Administration regulations.

### 2.2. Demographic Variables

Demographic information was obtained by NHANES staff through screening interviews at home. Various questionnaires were administered to gather data at the individual, family, and household levels [23]. Demographic variables included in this study were age, sex, race and ethnicity of the child, the household reference (HR) person’s age, sex, education, and marital status, and family income to poverty ratio (PIR). PIR was calculated by dividing family income by the poverty guidelines for the survey year. Poverty guidelines vary by family size and geographic location [24]. For this study, PIR was used to create two categories of income status, low (PIR < 1.3) and mid-high (PIR ≥ 1.3), as an indication of socioeconomic status based on eligibility for receiving benefits via the Supplemental Nutrition Assistance Program (SNAP). In the U.S., incomes under 130% of the federal poverty level (i.e., PIR of 1.3) are required to receive SNAP benefits [25]. The HR person’s education level was categorized as less than high school, high school/General Educational Development (GED)/some college, and college graduate or above. The HR person’s marital status was categorized as married or living with a partner, widowed/divorced/separated, and never married. The HR person’s age was categorized as <20–40 years and 40–60+ years.

### 2.3. Dietary Intake

NHANES staff members collected dietary intake data through 24-h dietary recalls in the Mobile Examination Center (MEC) by private interviews with a proxy for 2–5-year-old children [21]. The 24-h recall consisted of questions regarding the type and amount of food or beverage the child had consumed in the past day. NHANES utilized the U.S. Department of Agriculture (USDA) Automated Multiple-Pass Method (AMPM) to ensure more accurate responses during the recall [26]. Recalls were considered reliable if the respondent completed the first four steps of the AMPM and if the reported foods and beverages were identified.

The total nutrient intakes based on the 2 days of recall data were used to estimate micronutrient intakes for nutrients of concern such as calcium, iron, vitamin D, vitamin E, potassium, and sodium. Intakes of food group components were obtained from the Food Patterns Equivalents Database (FPED), which is derived from the dietary recall interview data of NHANES and reports on food group categories [27]. This study evaluated the intakes of total fruit, total vegetables, whole grains, total dairy, oils, saturated fatty acids, and added sugars. The total Healthy Eating Index (HEI)-2015 score was calculated using the National Institutes of Health, National Cancer Institute Statistical Analysis System (SAS) code [28] to measure diet quality based on alignment with the 2015–2020 Dietary Guidelines for Americans [29]. The maximum total score is 100.

### 2.4. Body Mass Index

Anthropometric data were collected in the MEC by trained NHANES staff according to standard procedures [30]. For 2–5-year-olds, measurements included weight and standing height. Weight was measured in kilograms using a high performance digital weight scale built into the exam room floor and linked directly to the NHANES Integrated Survey Information System (ISIS) anthropometry application (Mettler Toledo ICS469). Standing height was measured with a digital stadiometer connected to an acrylic headpiece and interfaced directly with the ISIS anthropometry application. Body mass index (BMI) was calculated by dividing the participant’s weight in kilograms by height in meters squared. Four BMI categories were created for 2–5-year-olds based on the CDC sex-specific 2000 BMI-for-age growth charts [31]. The categories included underweight (BMI < 5th percentile), normal weight (BMI 5th to <85th percentile), overweight (BMI 85th to <95th percentile), and obese (BMI ≥ 95th percentile).

### 2.5. Data Analysis

Data from the NHANES datasets were analyzed using SAS Survey statistical software (version 9.4) (SAS Analytic Software, Cary, NC, USA). The SAS Surveyfreq procedure was used to describe categorical variables for all children and by PIR categories (low vs. mid-high), such as demographic data for children, BMI percentile categories, and HR person’s characteristics. The Rao-Scott Chi Square test was used to determine differences in categorical variables by PIR categories. SAS Surveymeans was used to determine the mean dietary intakes and standard errors of micronutrients and food groups for all children and by PIR categories. The SAS Surveyreg procedure was used to perform linear regression analyses for all micronutrients, food group intakes, and the total HEI-2015 score based on PIR level adjusted for child age and sex, race and ethnicity, and energy intake (except for the total HEI-2015 score model), and HR persons’ sex and marital status. Model statements in the SAS Surveyreg procedure specified dependent variables (intakes of micronutrients and food groups, and HEI-2015 total score) as a function of the independent variable (PIR level) [32]. Analyses were not additionally adjusted for household or child food security, receiving WIC benefits in the past 12 months, or HR person’s education because these values were highly correlated with PIR. Dietary intakes were not normally distributed. Therefore, values were log transformed and used in the analysis. For ease in interpretation, non-transformed values were presented in tables. A *p*-value < 0.05 was considered statistically significant with Bonferroni Holm corrections applied for multiple comparisons across the nutrient intakes and food group data. Appropriate weight statements constructed based on combined survey cycles were used in the analyses to account for the complex, multistage probability sampling design to make estimates that were representative of the U.S. civilian non-institutionalized population.

## 3. Results

### 3.1. Demographic Characteristics

Children (*n* = 2249) were divided into a low PIR group (<1.3) (*n* = 1145, 41.4%) and a mid-high PIR group (≥1.3) (*n* = 1104, 58.6%). Children were somewhat evenly divided by year of age and sex, with no differences by low vs. mid-high PIR level (Table 1). Mexican American/Other Hispanic, Non-Hispanic Black, and Non-Hispanic Asian children were more likely to have a low vs. mid-high PIR level compared to Non-Hispanic White children (*p* < 0.0001). Female HR persons (*p* = 0.0003), HR persons who were single (*p* < 0.0001), and those with less education (*p* < 0.0001) were more likely to have low vs. mid-high PIR level compared to their counterparts. No differences were observed in BMI classes by PIR level (*p* = 0.291) with 73.6% of all children categorized into the normal weight category. About one-fourth of children (26.7%) in the low PIR group had marginal/low/very low child food security and 58.5% had marginal/low/very low household food security. Slightly more than half in the low PIR group (56.4%) received WIC benefits in the past year compared to 14.5% of those children in the mid-high PIR group.

### 3.2. Nutrient Intake

Children with low PIR level had lower dietary fiber (*p* = 0.003) and higher iron (*p* = 0.0003) intakes and marginally lower calcium intakes (*p* = 0.008) compared to children with mid-high PIR level (Table 2 and Table 3). Intakes of other micronutrients of concern did not differ by PIR levels.

### 3.3. Food Group Intakes

Intake of whole grain foods was significantly lower (*p* = 0.004) and intake of total fruit (*p* = 0.007) was marginally lower among children with low vs. mid-high PIR levels. Total dairy intake was lower (1.90 vs. 2.07 cup equivalents) among those with low vs. mid-high PIR level, but the difference was not statistically significant (*p* = 0.026). Intakes of total vegetables, total protein foods, and oils were also not different by PIR level. Total HEI-2015 scores were lower (*p* = 0.004) for children with low PIR vs. mid-high PIR levels.

## 4. Discussion

Intakes of several nutrients and food groups and total HEI-2015 scores were lower among young children (2–5 years) in low PIR vs. mid-high PIR groups. This finding was consistent with a previous study based on NHANES 2009–2014 data [33], which showed that for children 2–5 years, a lower total HEI-2015 score was observed for those children in families with PIR < 1 vs. PIR ≥ 1 (mean of 57.8 vs. 60.8). However, a recent study using 2013–2016 NHANES data did not report differences in intakes of food groups and components to limit by three PIR levels for children ages 2–8 years [7]. These results may differ from the current study based on examining usual intakes vs. 2 days of dietary recalls, inclusion of children 6–8 years, and use of different statistical analysis methods.

Children in the low PIR group in the current study had higher iron intakes compared to children in the mid-high PIR group, which could be explained by the tendency for higher intakes of protein foods among those in the low PIR group. Results from the Feeding Infants and Toddlers Study 2016 indicated that the risk of iron deficiency was lower among toddlers (6–23.9 months) in WIC-participating low-income households compared to those in non-WIC-participating, high-income households [20]. A similar association, although not statistically significant, was observed among preschoolers aged 24–47.9 months in the same study [20]. The percentage of children receiving WIC benefits in the past year based on the NHANES 2011–2018 data used for the current study was 56%, indicating that a proportion of the current sample population could have received WIC support. Federal supports such as WIC might be effective in reducing risk of iron deficiency among preschoolers in low-income households.

In the current study, calcium intake was marginally lower among children in the low PIR vs. mid-high PIR group. Total dairy intake also tended to be lower among children in the low PIR group, which may have contributed to lower calcium intake. Among non-WIC participants in another study based on NHANES 2005–2010, lower vs. higher income preschool children consumed less milk and more soda and fruit drinks [34]. Increasing milk intake among low-income children may improve calcium intake because milk was the primary food source of calcium intake among children 2–5 years [8]. A key benefit identified in a review on the effect of dairy intake in preschool children was the positive association between dairy intake and linear growth [35]. To provide consistent messaging for public health practitioners regarding the benefits of milk consumption by young children, a Consensus Statement by key U.S. health and nutrition organizations recognized milk as a healthy beverage choice for all preschool children [36].

Nutrients to limit in preschool children’s diets were not associated with PIR group in the current study, including sodium, added sugars, and saturated fats, indicating that the moderation components of the HEI-2015 may have been less important in contributing to the associations between the total HEI-2015 score and PIR group than adequacy components such as fruit and whole grain intakes. These findings are contrary to studies that have examined types of foods purchased by income. For example, among households participating in WIC, grocery store scanner data showed that about half (48%) of refreshment beverage purchases were accounted for by sugar-sweetened beverages [15]. Sugar-sweetened beverages accounted for a larger proportion of grocery dollars/week among lower compared to higher income participants in another study based on grocery receipts [19]. Lower income participants also had lower HEI-2010 empty calorie component scores compared to higher income participants [19]. Additional study of foods purchased and available for consumption by lower vs. higher income preschool children that are high in sodium, added sugars, and saturated fats may improve understanding of the role of these foods in preschool diet quality.

Dietary fiber intake was significantly lower among children in the low PIR group in the current study, which could be related to lower intakes of total fruit and whole grains compared to children in the mid-high PIR group. Fruit was one of the top food sources that contributed to fiber intake among children (2–5 years), along with bread products, and ready-to-eat cereals [8]. Differences regarding the amount and type of fiber in these food sources may have influenced dietary fiber intake. Fruit juice intake, separate from total fruit intake, was not significantly associated with PIR group, indicating that juice consumption may not have played a role in the lower fiber intake among children in the low PIR group. In addition to increasing fruit intake, choosing whole grain foods instead of refined grain foods could mitigate disproportionally lower fiber intakes among children in the low PIR group. Similarly, in another study based on NHANES 2009–2014 data, whole grain HEI-2015 component scores were 2.6 vs. 3.6/10 among children 2–5 years in families with PIR < 1 vs. PIR ≥ 1 [33]. Another study of WIC participants (1–4 years) in New York State showed that a majority had any daily vegetable (81%) and whole grain intake (65%) [37], indicating that WIC benefits may be related to increased availability of whole-grain products and consumption [38]. Therefore, purchasing whole grain foods and fruit high in fiber with WIC benefits could be encouraged to increase fiber intake among low-income children.

Results from the current study for all children are generally consistent with other studies indicating that a high proportion of children 2–5 years underconsume vegetables, whole grain foods and oils while overconsuming sodium, added sugars and saturated fats [3,4,5,6,7]. In addition, a longitudinal analysis of dietary intakes among 3-year-old non-Hispanic African American or White children from 3-day food diaries in a Midwestern U.S. city also showed a need to improve fruit, vegetable and whole grain intake and reduce intake of solid fats and added sugars [39]. The total HEI-2015 score for all children in the current study was 56/100, in line with other studies indicating a need for improvement in food group intakes among U.S. preschool children, regardless of PIR group.

This study includes several strengths and limitations. Strengths were the use of nationally representative U.S. data for children and two days of reliable dietary intake data using the standardized AMPM method by trained staff to reduce measurement error [40]. However, accuracy of dietary intake data may have been affected by response biases based on proxy reports for children 2–5 years of age. Many preschool children spend time in childcare settings where the proxy reporter may not be present to observe child eating occasions. Proxy reporters may over or underestimate intake based on the perceived healthfulness of foods consumed by the child. Estimating portion sizes accurately may also present challenges for proxy reporters. In addition, supplement consumption was not considered, which could have resulted in underestimation of nutrient intakes. Lastly, the same poverty guidelines used in NHANES 2011–2018 were established for the 48 contiguous states and the District of Columbia [41], which might not reflect the poverty situation in each state.

## 5. Conclusions

In this study based on nationally representative data, marginally lower calcium, and lower fiber intakes, lower overall HEI-2015 scores, and lower intakes of fruits and whole grain foods were observed among children aged 2–5 years in lower PIR households compared to mid-high PIR households. Increasing consumption of milk, fruit, and whole grains may improve calcium and fiber consumption and total HEI-2015 scores. Participation in the WIC program could mitigate disproportionately lower nutrient intakes of preschool children in low-income households through enhanced availability of fruit and whole grain foods. Future research could be directed to public health interventions for low-income families that are tailored to specifically focus on dietary components found to be consumed at lower levels by the low PIR vs. mid-high PIR group in the current study.

## Figures and Tables

**Table 1:** Demographic characteristics of children 2–5 years (with 2 days reliable dietary intake data) and household reference persons, NHANES (2011–2018).

	All *n* (% ^2^)	PIR ^1^ < 1.3 *n* = 1145 *n* (% ^2^)	PIR ≥ 1.3 *n* = 1104 *n* (% ^2^)	*p*-Value ^3^
Child age	*n* = 2249	*n* = 1145	*n* = 1104	0.147
2 years	707 (25.0)	367 (25.8)	340 (24.4)	
3 years	520 (25.9)	255 (23.8)	265 (27.4)	
4 years	521 (24.9)	266 (24.5)	255 (25.2)	
5 years	501 (24.2)	257 (25.9)	244 (23.0)	
Child sex	*n* = 2249	*n* = 1145	*n* = 1104	
Male	1118 (49.3)	572 (50.3)	546 (48.6)	0.449
Female	1131 (50.7)	573 (49.7)	558 (51.4)	
Child race/ethnicity	*n* = 2249	*n* = 1145	*n* = 1104	<0.0001
Mexican American/Other Hispanic	671 (24.9)	412 (36.5)	259 (16.6)	
Non-Hispanic White	641 (52.2)	230 (34.4)	411 (64.8)	
Non-Hispanic Black	555 (13.0)	384 (21.2)	171 (7.2)	
Non-Hispanic Asian	207 (4.4)	53 (2.6)	154 (0.8)	
Other race—including multi-racial	175 (5.6)	66 (5.3)	109 (5.8)	
Child BMI category (*n* = 525 missing)	*n* = 1724	*n* = 905	*n* = 819	0.291
Underweight	68 (3.0)	33 (3.1)	35 (3.0)	
Normal weight	1240 (73.6)	628 (70.8)	612 (75.7)	
Overweight	224 (13.2)	130 (14.4)	94 (12.4)	
Obese	192 (10.1)	114 (11.7)	78 (9.0)	
Child food security (*n* = 44 missing)	*n* = 2205	*n* = 1104	*n* = 1101	<0.0001
Full or marginal	1815 (84.6)	817 (74.3)	998 (91.5)	
Marginal/low/very low	390 (15.4)	287 (26.7)	103 (8.5)	
WIC benefit last 12 months (*n* = 44 missing)	*n* = 2205	*n* = 1104	*n* = 1101	<0.0001
Yes	867 (31.4)	638 (56.4)	229 (14.5)	
No	1338 (68.6)	466 (43.6)	872 (85.5)	
HR ^1^ person’s sex	*n* = 2249	*n* = 1145	*n* = 1104	0.0003
Male	1004 (50.0)	412 (42.1)	592 (55.6)	
Female	1245 (50.0)	733 (57.9)	512 (44.4)	
HR person’s age	*n* = 2249	*n* = 1145	*n* = 1104	0.881
<20–40 years	1628 (73.9)	844 (73.2)	784 (72.7)	
40–60+ years	621 (27.1))	301 (26.8)	320 (27.3)	
HR person’s marital status	*n* = 2203	*n* = 1104	*n* = 1099	<0.0001
Married/living with partner	1655 (81.4)	687 (67.2)	968 (91.2)	
Widowed/divorced/separated	306 (11.9)	222 (20.1)	84 (6.2)	
Never married	242 (6.8)	195 (12.7)	47 (2.6)	
HR person’s education	2169	*n* = 1091	*n* = 1078	<0.0001
<HS ^1^	192 (7.4)	156 (14.1)	36 (2.8)	
HS/GED ^1^, some college	1553 (67.7)	873 (79.3)	680 (59.9)	
College grad or above	424 (24.9)	62 (6.6)	362 (37.3)	
Household food security (*n* = 44 missing)	*n* = 2205	*n* = 1104	*n* = 1101	<0.0001
Full	1254 (62.7)	439 (41.5)	815 (77.1)	
Marginal/low/very low	951 (37.3)	665 (58.5)	286 (22.9)	

^1^ PIR—Family income to poverty ratio, HR—household reference, HS—high school, GED—General Educational Development, ^2^ Weighted column percentages, ^3^ *p*-values based on Rao-Scott Chi-Square tests, *p* < 0.05.

**Table 2:** Nutrient intakes of children 2–5 years, NHANES (2011–2018) by family income to poverty ratio levels.

Nutrients ^1^	All *n* = 2249	PIR ^2^ < 1.3 *n* = 1145	PIR ≥ 1.3 *n* = 1104	
**To increase**	Mean (SE ^2^)	LS Mean ^2^ (SE)	LS Mean (SE)	*p* Value ^3^
Calcium (mg)	940 (13)	892 (15)	951 (15)	0.008
Iron (mg)	11.2 (0.1)	11.5 (0.2)	10.7 (0.1)	**0.0003**
Vitamin D (D2 + D3) (mcg)	5.8 (0.1)	5.7 (0.1)	5.7 (0.1)	0.517
Vitamin E (alpha-tocopherol) (mg)	5.6 (0.1)	5.5 (0.1)	5.6 (0.1)	0.116
Potassium (mg)	1956 (21)	1917 (20)	1954 (20)	0.207
Dietary fiber (g)	12.1 (0.1)	11.4 (0.2)	12.3 (0.2)	**0.003**
**To limit**				
Sodium (mg)	2229 (25)	2247 (22)	2174 (22)	0.108
Saturated fatty acids (gm)	19.7 (0.3)	19.5 (0.3)	19.5 (0.3)	0.893
Added sugars (tsp eq)	11.0 (0.2)	10.8 (0.3)	10.8 (0.3)	0.657

^1^ Nutrient data from dietary interview, total nutrient intakes, first and second days, added sugars data from FPED database food group data, ^2^ PIR—Family income to poverty ratio, SE—standard error, LS—least squares mean, ^3^ Regression models adjusted for child age, sex, race and ethnicity, and energy intake, and HR (household reference) persons’ sex and marital status, with Bonferroni Holm correction for multiple comparisons, bolded *p*-values indicate statistical significance.

**Table 3:** Food group intakes and diet quality scores of children 2–5 years, NHANES (2011–2018) by family income to poverty ratio levels.

Food Groups ^1^ and HEI-2015 Score	All *n* = 2249	PIR ^2^ < 1.3 *n* = 1145	PIR ≥ 1.3 *n* = 1104	
	Mean (SE ^2^)	LS Mean ^2^ (SE)	LS Mean (SE)	*p* Value ^3^
Total fruit (cup eq ^2^)	1.42 (0.04)	1.29 (0.04)	1.51 (0.05)	**0.007**
Fruit juice (cup eq)	0.54 (0.03)	0.58 (0.03	0.52 (0.04)	0.495
Total vegetables (cup eq)	0.66 (0.02)	0.67 (0.06)	0.65 (0.07)	0.630
Whole grains (oz eq ^2^)	0.77 (0.03)	0.69 (0.10)	0.84 (0.12)	**0.004**
Total protein foods (oz eq)	2.97 (0.05)	3.08 (0.07)	2.88 (0.07)	0.098
Total dairy (cup eq)	2.04 (0.04)	1.90 (0.05)	2.07 (0.06)	0.026
Oils (g)	16.1 (0.3)	15.9 (0.4)	15.9 (0.4)	0.836
HEI-2015 total score	55.9 (0.4)	54.4 (0.6)	57.2 (0.5)	**0.004**

^1^ Total fruit = total intact fruits (whole or cut) and fruit juices, total vegetable = total dark green, red and orange, starchy, and other vegetables; excludes legumes, whole grains = defined as whole grains and contain the entire grain kernel, total dairy = total milk, yogurt, cheese, and whey, oils = fats in nuts, seeds, and seafood; all unhydrogenated vegetable oils, fat present in avocado and olives, 50% of fat in margarines, ^2^ PIR—Family income to poverty ratio, SE—standard error, LS Mean—least squares mean, cup eq = cup equivalent, oz eq = ounce equivalent, ^3^ Regression models adjusted for child age, sex, race/ethnicity, and energy intake, and HR persons’ sex and marital status, with Bonferroni Holm correction for multiple comparisons, bolded *p*-values indicate statistical significance.

## Data Availability

Data supporting reported results can be found in publicly archived datasets at https://wwwn.cdc.gov/nchs/nhanes/default.aspx (accessed on 12 November 2021).
